# DeepASDPred: a CNN-LSTM-based deep learning method for Autism spectrum disorders risk RNA identification

**DOI:** 10.1186/s12859-023-05378-x

**Published:** 2023-06-22

**Authors:** Yongxian Fan, Hui Xiong, Guicong Sun

**Affiliations:** School of Computer Science and Information Security, Guilin University of Electronic Technology, Guilin, 541004 China

**Keywords:** ASD risk RNA, Deep learning, K-mer feature extraction, DeepASDPred

## Abstract

**Background:**

Autism spectrum disorders (ASD) are a group of neurodevelopmental disorders characterized by difficulty communicating with society and others, behavioral difficulties, and a brain that processes information differently than normal. Genetics has a strong impact on ASD associated with early onset and distinctive signs. Currently, all known ASD risk genes are able to encode proteins, and some de novo mutations disrupting protein-coding genes have been demonstrated to cause ASD. Next-generation sequencing technology enables high-throughput identification of ASD risk RNAs. However, these efforts are time-consuming and expensive, so an efficient computational model for ASD risk gene prediction is necessary.

**Results:**

In this study, we propose DeepASDPerd, a predictor for ASD risk RNA based on deep learning. Firstly, we use K-mer to feature encode the RNA transcript sequences, and then fuse them with corresponding gene expression values to construct a feature matrix. After combining chi-square test and logistic regression to select the best feature subset, we input them into a binary classification prediction model constructed by convolutional neural network and long short-term memory for training and classification. The results of the tenfold cross-validation proved our method outperformed the state-of-the-art methods. Dataset and source code are available at https://github.com/Onebear-X/DeepASDPred is freely available.

**Conclusions:**

Our experimental results show that DeepASDPred has outstanding performance in identifying ASD risk RNA genes.

## Background

Autism spectrum disorders (ASD) are neurodevelopmental disorders encompassed three types: autism, Asperger's syndrome, and pervasive developmental disorder to be classified. The main manifestations of ASD are difficulties with social and other interactions, communication, behavioral difficulties and the brain processing information in a different way than normal. ASD is heritable with a complex and heterogeneous genetic component and usually develops in the first three years of life [1–3]. At present, all known ASD risk genes are capable of encoding proteins, and a number of de novo mutations disrupting protein-coding genes have been shown to cause ASD [4–6]. A growing volume of research indicates that RNA plays an important role in the translation of biological genetic information [7], RNA modifications are associated with multiple diseases in organisms [8]. Therefore, it is important to explore RNA-based classification prediction for the treatment of ASD. For the diagnosis of ASD, several clinical information of ASD patients, such as symptom data, magnetic resonance image data and whole brain structural image data, are usually relied upon to build computational prediction models [9–12]. However, these models are not applicable to the prediction of ASD risk gene. In addition, genetic methods for identifying ASD risk gene, such as genome-wide association studies, copy number variation studies, and whole exome sequencing, are time-consuming and laborious. Therefore, there is a need to develop more efficient computational methods or tools.

To date, there have been a number of studies that have used machine learning to target ASD with RNA, and these studies have yielded some results. In 2016, Cogill et al. used wrapper and best-first search methods for feature selection and constructed support vector machine (SVM) models based on brain development gene expression data [13]. In 2018, Gok et al. used Haar wavelet transform to extract features on gene expression values and combined with Bayes network for classification and prediction of ASD risk gene [14]. In 2020, Wang et al. utilized a autoencoder network for representation learning of gene expression data, followed by a random forest network-derived K-mer method for feature representation of gene transcript sequences, and finally three machine learning models, including logistic regression (LR), SVM and random forest (RF), combined with ten-fold cross-validation were used to predict and rank RNA sequences, respectively, and RF was selected as the final model [15]. Zhao et al. developed the random walk method based on AutDB for predicting genes associated with ASD [16]. 2021, Hasan et al. collected 1055 data from toddlers and 705 data from adults by Q&A, including age, family history of ASD, and app used, and further used machine learning methods to predict whether they had ASD or not[17]. Lin et al. proposed the ASD-Risk method using inheritable bi-objective combinatorial genetic algorithm and SVM to further improve the prediction performance for ASD risk gene [18]. Although ASD-Risk has been improved compared to existing studies, it still suffers from low accuracy and weak model generalization.

**Fig. 1:**
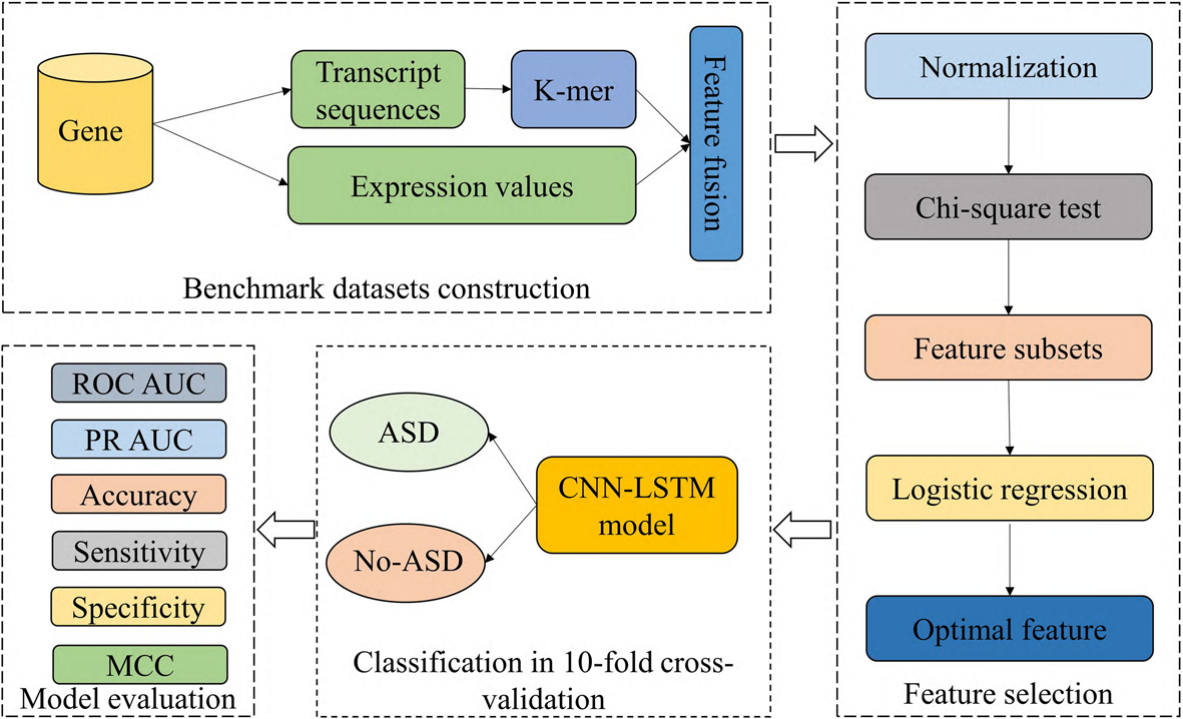
The framework of DeepASDPred for identifying ASD-Risk RNA gene

In this work, we proposed a new computational method DeepASDPred to identify ASD risk gene, and the core classification module is a convolutional neural network (CNN) and long short-term memory (LSTM) parallel concatenated model. First, we converted the original RNA nucleotide sequences into vector form using K-mer. Then, the vector features were fused with their corresponding gene expression values. To reduce the redundancy of features and to speed up the computation, we further performed feature selection on the fused features. Finally, the optimized features were transferred to deep learning models based on CNN and LSTM to classify RNA genes. Based on tenfold cross-validation, we used robust metrics the area under the receiver operating characteristic curve (ROC AUC) and the area under the Precision-Recall curve (PR AUC) for model performance evaluation and comparison [19, 20], and the flowchart of DeepASDPred is shown in Fig. 1.

## Results and discussion

### CNN parameters selection

The appropriate CNN parameters have a significant impact on the model prediction performance. Using the best subset of features as input, we constructed a predictor based on one-dimensional convolutional neural network. We experimented to obtain the optimal values of the parameters based on the range of parameters given in Table 1. Based on the results of the tenfold cross-validation, we obtained the optimal model structure, and the selected parameters are listed in Table 1.

**Table 1:** Details of tuning parameters in CNN

Parameters	Range	Optimal parameters
Convolutional Layer	[1–3]	1
Filter	[16, 32, 64]	64
Kernel_size	[3, 5, 7, 9]	3
Stride	[1–3]	1
Learning_rate	[1e−6,1e−2]	1e−4
Batch_size	[32, 64, 128, 256]	64

### Comparison of different model structures

**Fig. 2:**
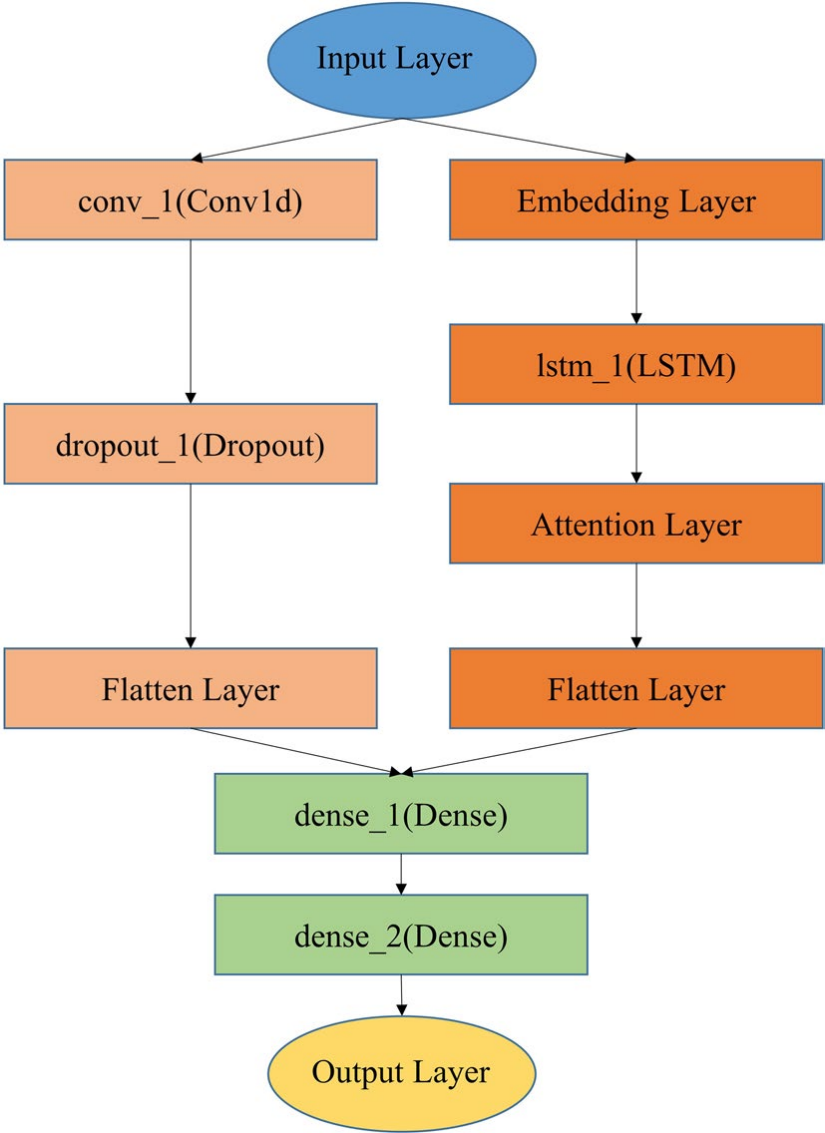
The model structure of CNN-LSTM

**Fig. 3:**
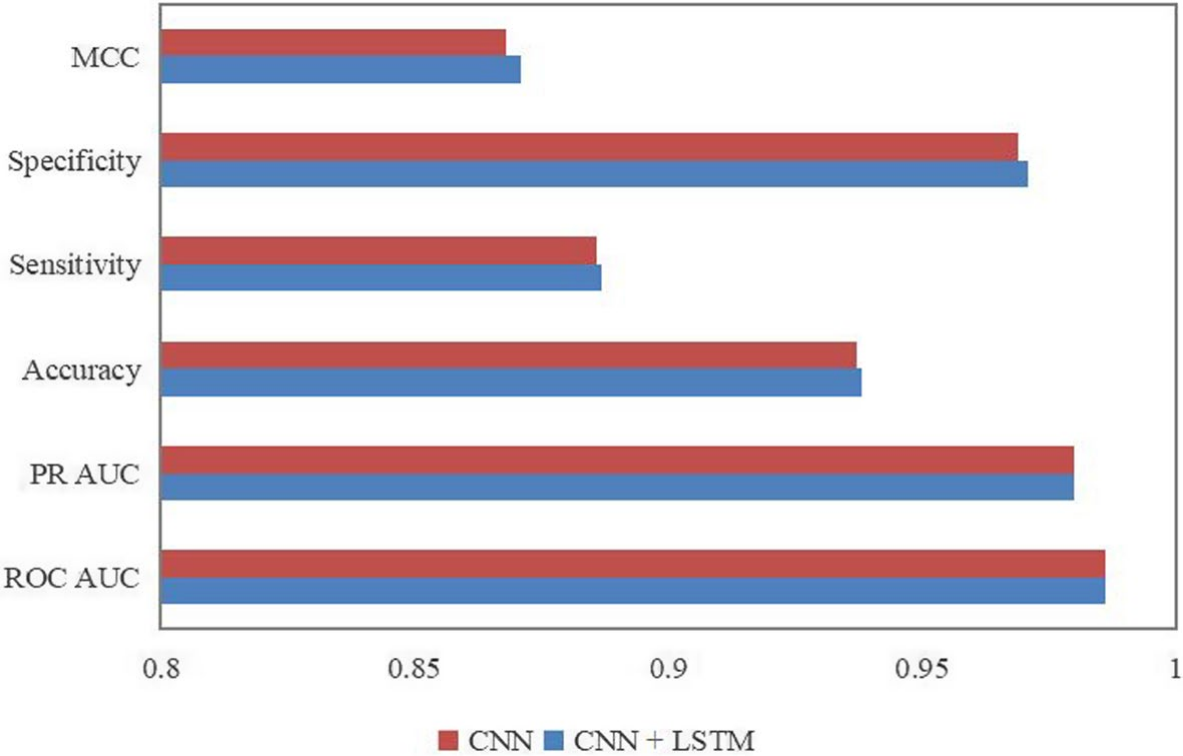
Performance comparison between CNN and CNN-LSTM

Above the excellent performance of the CNN model, we added LSTM to extend the model to obtain even better performance. Referring to Tang’s work [21], another part of LSTM was increased in parallel with the CNN module, and the detailed structure of the model is shown in Fig. 2. Comparing with CNN classification separately, the CNN-LSTM model showed a slight improvement in Accuracy, Mathews correlation coefficient (MCC), and other metrics in Fig. 3. More specifically, the six evaluation metrics obtained with the CNN-LSTM model: ROC AUC, PR AUC, Accuracy, Sensitivity, Specificity and MCC are 0.986, 0.981, 0.937, 0.882, 0.971 and 0.867, respectively. Therefore, we chose the CNN-LSTM as the final training model.

### Comparison of different feature selection methods

**Fig. 4:**
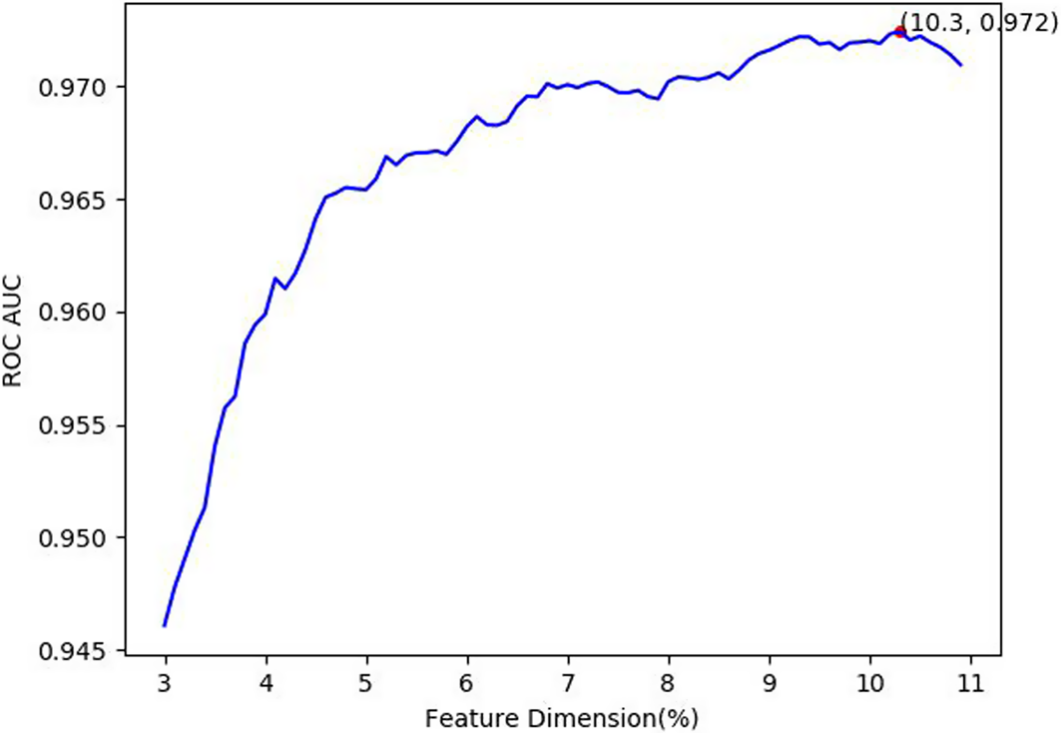
Feature dimension selection of chi-square test and LR

**Fig. 5:**
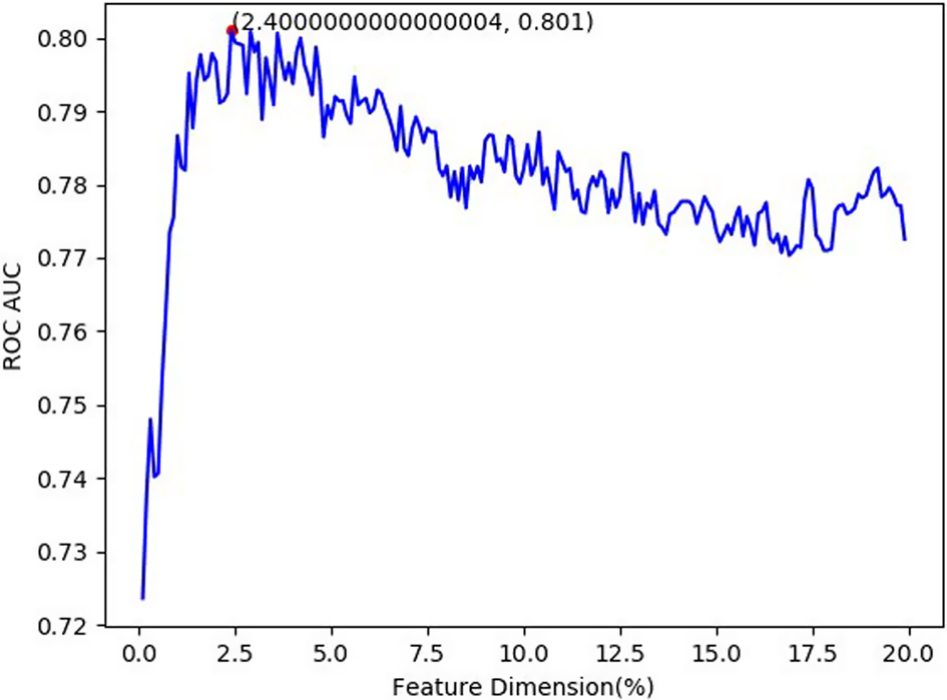
Feature dimension selection of chi-square test and XGboost

Apart from employing the feature selection method with chi-square and LR, we also applied XGboost for feature selection replacing LR. As shown in Fig. 4, the highest ROC AUC was achieved when the feature dimension was 10.3% with a value of 0.972 by the chi-square test combined with LR method. In Fig. 5, the highest AUC was obtained when the feature dimension was 2.4% using chi-square test and XGboost for feature dimension selection. And the latter was significantly worse than the former. The result indicates our feature selection scheme is reasonable and effective.

### Comparison with machine learning algorithms

**Fig. 6:**
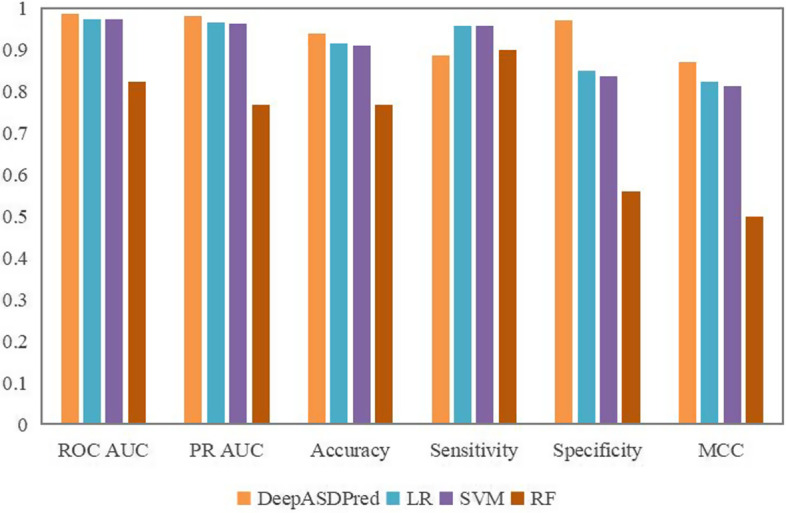
Performance comparison between DeepASDPred and single classifiers

As well as using deep learning methods, we also tried several traditional single classification algorithms, including RF, SVM, and LR. We used GridSearch to find the optimal parameters and obtained performance evaluation metric results using tenfold cross-validation. Figure 6 shows the gaps between the three single classifiers and our method. The results show our model achieved the best performance, LR and SVM are relatively better, and RF performs the poorest.

### Comparison of different feature representation performances

**Fig. 7:**
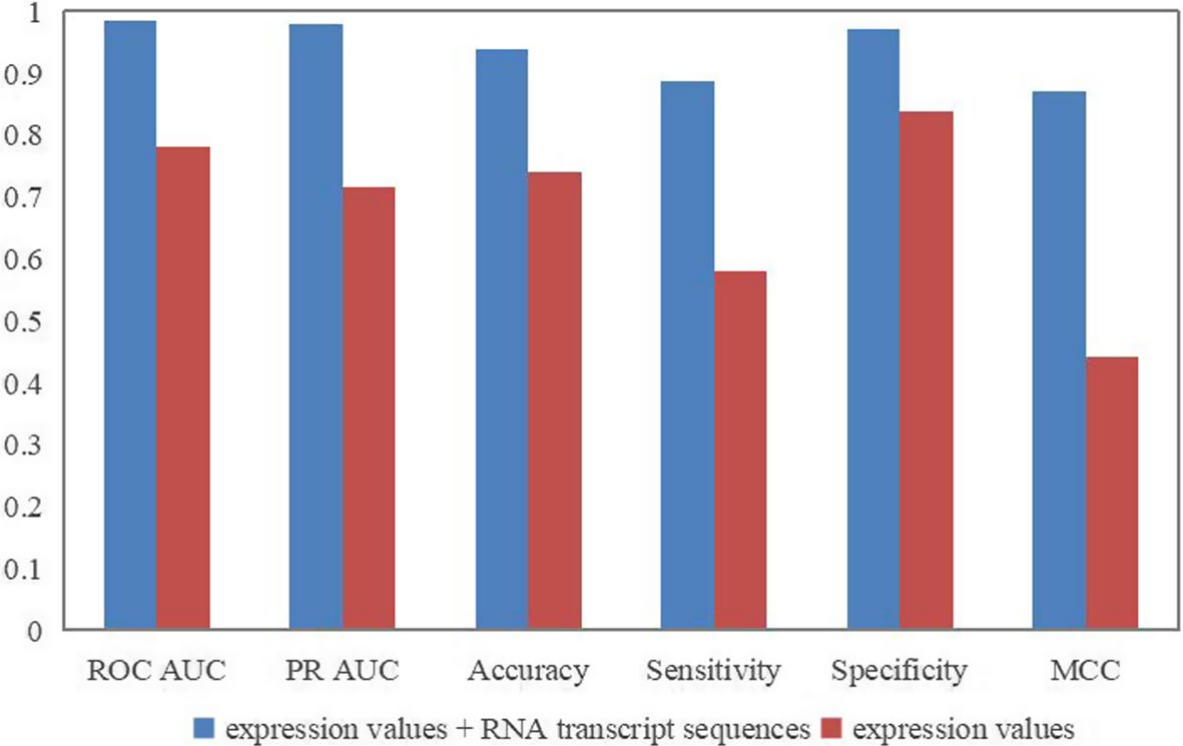
Comparison of model performance with different feature representations

In this task, we used two types of data, gene expression values and RNA transcript sequences. Although ASD has severe genetic heterogeneity, it is not yet known whether ASD risk gene shares common nucleotide sequence features [22]. Therefore, the reference value of the nucleotide sequence is probably inferior to the gene expression value. We compared the gene expression value as a single feature and the both as features at the same time. The results are shown in Fig. 7, showing the both are better than the single and validating the superiority feature representation of DeepASDPred.

### Comparison with state-of-the-art methods

In this part, we compared DeepASDPred with state-of-the-art methods. To the best of our knowledge, several prediction methods have been proposed in recent years regarding prediction of RNA associated with ASD. We selected four methods to compare with DeepASDPred, with the first, second, and fourth methods having the same dataset and the third method updating the former dataset, and we updated it again on the dataset applied by the third method. We recorded the average value of the tenfold cross-validation for the evaluation comparison. The results are shown in Table 2, it can be seen DeepASDPred has been significantly improved in all performance metrics, showing the excellence of our proposed method.

**Table 2:** Comparison of performance with state-of-the-art methods

Methods	Accuracy	Sensitivity	Specificity	MCC
Wang’s SVM (2016)	0.767	0.744	0.772	0.419
Murat’s Bayes (2018)	0.783	0.902	0.665	0.583
Wang’s RF (2020)	0.770	0.698	0.799	0.471
Lin’s ASD-Risk (2021)	0.818	0.840	0.790	0.630
DeepASDPred	**0.938**	**0.887**	**0.971**	**0.871**

## Methods

### Benchmark dataset

A reliable benchmark dataset is necessary to construct stable and effective computational models. In this work, we followed the dataset used by Wang’s study[15]. To be specific, we used RNA as instances and integrated gene expression values and sequence information as features to form the benchmark dataset. In addition, an increasing number of studies have identified some new ASD risk genes, and some genes previously identified as unrelated to ASD have been later shown to be associated with ASD. Due to these considerations, we updated the baseline dataset to include 1005 positive samples and 1590 negative samples. The positive samples are from the Simons Foundation Autism Research Initiative Gene database [23], and the negative samples are disease genes not associated with ASD, and the details of the dataset are shown in Table 3.

**Table 3:** Details of the dataset for predicting RNA genes associated with ASD

	ASD	No-ASD
Raw	1005	1590
Nucleotides	[413, 21103]	[420, 13203]
After K-mer	4^8^	4^8^
Expression values	524	524

### Gene expression values and RNA transcript sequences

We obtained the gene expression values of RNA from the BrainSpan Atlas of the Developing Human Brain (https://www.brainspan.org). BrainSpan provides a publicly available human developmental transcriptome dataset including 524 samples from 26 brain structures with developmental time points ranging from 8 weeks to 40 years [24]. Gene expression values are expressed as reads per kilobase of transcript per million mapped reads (RPKM). For computational convenience, the obtained data were ranged from 0 to 1 by max-minimum normalization.

We obtained the RNA transcript nucleotide sequences from the GENCODE FASTA file (GRCh38) (https://www.gencodegenes.org/human/) [25]. Subsequently, K-mer was used to encode the transcribed nucleotide sequences and normalize them by sequence length.

### Feature extraction and selection

In this section, we extracted and selected features from the raw sequence samples, and we represented the RNA transcript sequence as a sequence *D* of length *L*:

1 $$D = R_{1} R_{2} \ldots R_{i} \ldots R_{L}$$

where

2 $$R_{i} \in \left\{ {{\text{A}}({\text{adenine}}),{\text{C}}({\text{cytosine}}),{\text{G}}({\text{guanine}}),{\text{U}}({\text{uracil}})} \right\}$$

### K-mer nucleotide composition

Almost all existing machine learning algorithms can only deal with vectors rather than sequence samples. The reason is if raw sequences are used as training data, it is difficult to obtain a model that can cover all cases [26]. The pseudo amino acid composition (PseAAC) was firstly proposed by Chou et al. to calculate sequence-pattern information of proteins [27]. And with its influence, the pseudo k-tuple nucleotide composition (PseKNC) was created, via this method we can transform DNA or RNA sequences into feature vectors [28]. It has proved to be useful, especially after the "Pse-in-One" server release [29], allowing users to generate biological sequences into the required feature vectors for their research purposes. K-mer can be treated as a simple PseKNC, and it is an effective sequence representation method showing in various fields of sequence [30–32]. The implementation of K-mer can be described as:

1. Set a window of size *k*, i.e., there are 4^ k^ base combination forms, and then slide on the sequence D with a step size of 1. For each slide step, a short sequence of *k* is obtained;
2. Observe the number of occurrences of the i-th K-mer *β*_*i*_;
3. Finally, the K-mer feature vector of the sequence can be expressed as *V*:

3 $$V = \left[ {\varphi_{1} ,\varphi_{2} , \cdot \cdot \cdot ,\varphi_{i} , \cdot \cdot \cdot ,\varphi_{{4^{k} }} } \right]$$

where the frequency of the i-th *k*-mer *φ*_*i*_ can be expressed as:

4 $$\varphi_{i} = \frac{{\beta_{i} }}{{\sum\nolimits_{i = 1}^{{4^{k} }} {\beta_{i} } }} = \frac{{\beta_{i} }}{L - k + 1}$$

According to Su’s work and our experimental validation stated, the 8-mer distribution has a unique significance in the evolutionary mechanism of RNA[33]. Therefore, we set k = 8 to complete the coding of RNA sequence features, and the detailed information of the dataset after feature extracting is shown in Table 3.

### Feature selection

The data for RNA nucleotide sequences is huge after the completion of K-mer encoding and there may be a large amount of redundant information. In addition, a large amount of training data can lead to problems such as large computational effort, long training time and weak model migration ability during model construction. Therefore, a reasonable selection of the best feature subset is essential. In previous work, Wang et al. proposed PA-PseU to identify RNA pseudouridine sites [34]. In this section, we followed the way of PA-PseU for feature selection to reduce the dimensionality of features. PA-PseU utilizes the chi-square test and LR, where the chi-square test measures the independence between random variables and eliminates the features most likely to be independently classified; and logistic regression is employed as an effective linear classifier. PA-PseU can be partitioned into three steps:

1. Maximum-minimum normalization after merging gene expression values and sequence feature vectors;
2. The chi-square test scores are calculated according to the following formula:

5 $$\chi^{2} = \sum\limits_{i = 1}^{k} {\frac{{(A_{i} - np_{i} )^{2} }}{{np_{i} }}}$$

where is the frequency of the i-th observation in the feature vector, *k* is the total number of observations, is the expected frequency of the i-th observation, and n is the total number of samples. Then we rank the scores of each feature in descending order, with higher scores implying better classification, and subsequently set a filter with a range of 0.1%-20% and a step size of 0.1%, obtaining a feature subset for each step;

- (3)LR is applied to fit each feature subset, the L2 norm penalty is used to reduce the risk of overfitting, and the ROC AUC value of each feature subset is calculated using five-fold cross-validation to obtain the best feature subset.

### Convolutional neural network

Compared with traditional learning algorithms, CNN is a feed-forward neural network [35], and it shares weights through convolutional kernels and filters, remarkably reducing the complexity of the model. CNN is preferred by numerous researchers because of its powerful self-learning capability and superior parallel processing performance, especially in image learning. There are already many mature CNN-based models, like LeNet, VGG, ResNet, etc.

In general, a convolution module consists of two operations: convolution and pooling. Convolution (1D convolution for example) is performed by sliding a filter (with number *f* and size *s*) over the input matrix with stride of size *t*, and the filter is dotted with the input receptive field to acquire different feature maps by sharing the learnable parameters with input, and the multi-dimensional convolution enables to acquire different dimensional feature maps. The activation function is applied at the end of the convolution to increase the non-linear characteristics of the CNN, and the common activation functions including rectifed linear unit(ReLU), tanh, sigmoid and softmax. To improve the model training fitting speed, we chosen ReLU as the activation function for our model [36]. The formula for the convolution defines as follows:

6 $${\text{Conv}}(X) = {\text{ReLU}}\left( {\sum\limits_{s = 0}^{S - 1} {\sum\limits_{f = 0}^{F - 1} {WX} } } \right)$$

where *X* is the input matrix, *W* is the weight matrix of size *S* × *F*, the mathematical expression of ReLU as follows:

7 $${\text{ReLU = }}\left\{ {\begin{array}{*{20}l} {0,} \hfill & {{\text{if }}x < 0} \hfill \\ {x,} \hfill & {{\text{if }}x \ge 0} \hfill \\ \end{array} } \right.$$

The sotfmax activation function is added after the Dense layer to extract the correlation between the features. We use categorical_crossentropy as the loss function to get the final output of the model, and the loss function is calculated as follows:

8 $${\text{Loss}} = - \sum\limits_{i = 1}^{N} {y_{i} \cdot \log } y^{\prime}_{i}$$

where *y*_*i*_ denotes the label of sample i, *y*_*i*_^*'*^ is the positive predictive value of sample *i*, and *N* is the size of the sample.

The pooling is a non-linear down-sampling operation serving to reduce the space of the representation, the number of parameters in training, memory, etc., in addition to decreasing the risk of over-fitting.

### Long short-term memory

**Fig. 8:**
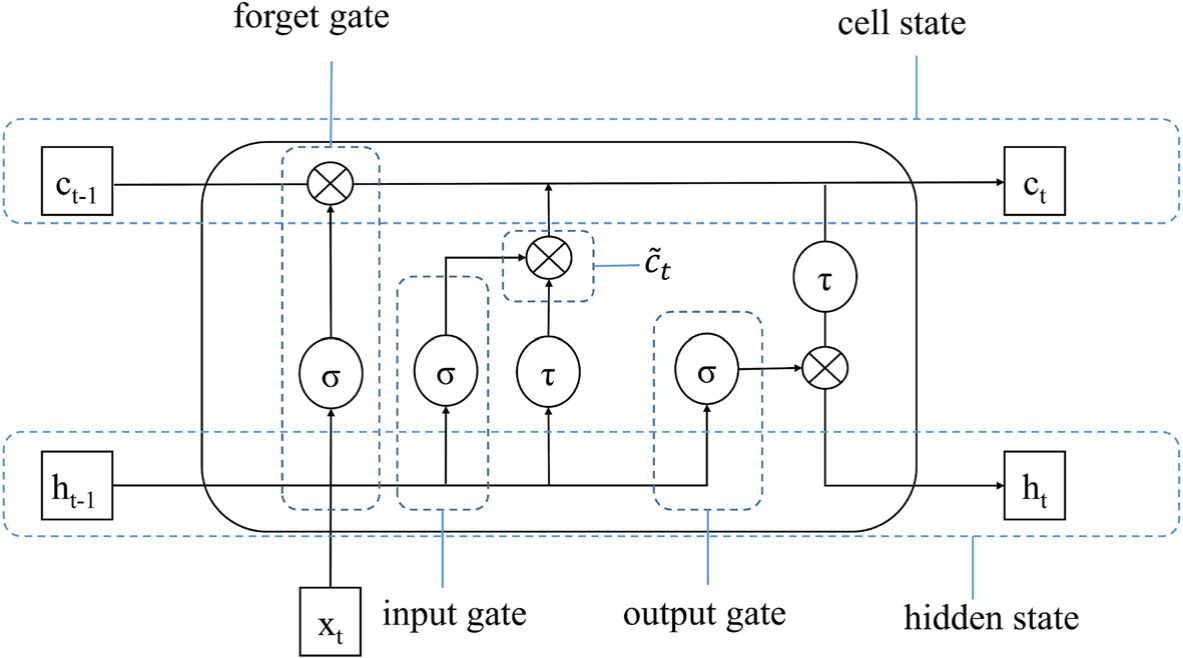
The structure diagram of LSTM

LSTM is an improved recurrent neural network (RNN) dedicated to processing sequence data [35, 37, 38]. LSTM effectively solves the gradient disappearance and gradient explosion problems of RNN in training long-term sequences and is able to accurately calculate the dependencies between words in a sequence, causing LSTM a rapid replacement for RNN in most application scenarios. The core module of LSTM is cell state, and LSTM determines the cell state through forget gate, input gate and output gate. The specific process is shown in Fig. 8. Suppose the present time is *t*, and *x*_*t*_ is the input at *t*, *h*_*t-1*_ is the output value of the last unit time hidden state. The calculation formula for the forget gate is:

9 $${\text{f}}_{t} = \sigma \left( {W_{f} \cdot [h_{t - 1} ,x_{t} ] + b_{f} } \right)$$

where *W*_*f*_, *b*_*f*_ are the weight matrix and bias of the forget gate, respectively, σ is the sigmoid funtion. After calculating by the above formula, if the result is 1, the output value of the last unit time cell state c_*t*-1_ will be retained, and the contrary will be forgotten. The input gate is calculated as:

10 $${\text{i}}_{t} = \sigma \left( {W_{i} \cdot [h_{t - 1} ,x_{t} ] + b_{i} } \right)$$

where *W*_*i*_, *b*_*i*_ are the weight matrix and bias of the input gate, respectively. Also calculated by the sigmoid function, the result decides which information will be updated. The output gate is calculated as:

11 $${\text{o}}_{t} = \sigma (W_{o} \cdot [h_{t - 1} ,x_{t} ] + b_{o} )$$

where *W*_*o*_, *b*_*o*_ are the weight matrix and bias of the output gate, respectively. The calculation of sigmoid function is involved in determining the value of the hidden state h_*t*_ at the current time *t*. Finally, h_*t*_ and the value of cell state at the current time *t* c_*t*_ are calculated as follows:

12 $${\tilde{\text{c}}}_{t} = \tanh \left( {W_{c} \cdot [h_{{t - 1}} ,x_{t} ] + b_{c} } \right)$$

13 $${\text{c}}_{t} = {\text{f}}_{t} \cdot {\text{c}}_{{t - 1}} + {\text{i}}_{t} \cdot {\tilde{\text{c}}}_{t}$$

14 $${\text{h}}_{t} = o_{t} \cdot \tanh \left( {{\text{c}}_{t} } \right)$$

where *W*_*c*_, and *b*_*c*_ are the weight matrix and bias of the cell state, respectively.

### Attention mechanism

Similar to the way people observe the scene, we pay different attention to different things. Attention is a mechanism assigning different weights to different positions of a sequence and has been a commonly module in deep learning since it was proposed [39]. We added a feed forward attention to the LSTM part of the model to solve the long-term dependency problem. The procedure can be described as follows [40]:

1. Calculate the weight value e_*t*_ of the hidden state in each time step of the LSTM with the following formula:
  15 $${\text{e}}_{t} = a({\text{h}}_{t} )$$
2. Normalize with softmax function:
  16 $${\uptheta }_{t} = \frac{{\exp (e_{t} )}}{{\sum {\exp (e_{t} )} }}$$
3. The final sum normalized weights of the hidden state are obtained as follows:
  17 $$c = \sum\limits_{i = 1}^{t} {\theta_{t} h_{t} }$$

### Cross validation and model evaluation

In this study, tenfold cross-validation was used to objectively evaluate the performance of our proposed method. To obtain reliable estimates of the prediction results, the following experiments were repeated 50 times and the average of all evaluation results were taken as the final model performance. We used Accuracy, Sensitivity, Specificity, and MCC as the evaluation metrics of the model. Their definitions are listed as follows:

18 $${\text{Accuracy = }}\frac{TP + TN}{{TP + TN + FP + FN}}$$

19 $${\text{Sensitivity = }}\frac{TP}{{TP + FN}}$$

20 $${\text{Specificity = }}\frac{TN}{{TN + FP}}$$

21 $${\text{MCC = }}\frac{TP \times TN - FP \times FN}{{\sqrt {\left( {TP + FP} \right)\left( {TP + FN} \right)\left( {TN + FP} \right)\left( {TN + FN} \right)} }}$$

where TP is the number of true positives; TN is the number of true negatives; FP is the number of false positives; and FN is the number of false negatives. In addition, ROC AUC and PR AUC were used as auxiliary measures of model performance. the ROC curve depicts the plot of true positive rate versus false positive rate at different model output thresholds, and the PR curve is the plot of precision versus sensitivity at different model output thresholds. The MCC, the ROC AUC and PR AUC are closer to 1 representing the better performance of the model.

## Conclusion

Approximately 24.8 million people had ASD worldwide in 2015, and even in developed countries in 2017, more than 1.5% of children were still clinically diagnosed with ASD [41]. Genetic prediction of relevant ASDs has been extensively studied, but there is still much room for performance improvement. In this work, we proposed a new method DeepASDPred for ASD risk gene identification. DeepASDPred is based on CNN and LSTM and only uses RNA nucleotide sequences and gene expression values as a benchmark dataset without biological prior knowledge. In particular, after encoding the data features, we utilized chi-square test and LR to select the best feature subset to reduce data redundancy and speed up training. In addition, we compared DeepASDPred with three single classifiers and state-of-the-art methods. The comparison results show DeepASDPred obtained the best performance and validate the efficient performance of DeepASDPred in identifying ASD risk gene.

Nevertheless, there is still some work needed to be further investigated in the future. Firstly, the gene expression values used for characterization data suffer from the drawback of small source sample data. In addition, recent studies suggest that non-coding RNAs may also have an impact on ASD [6]. Therefore, increasing the addition to the ASD-related gene database and expanding the exploration of non-coding RNAs have definite research value for the task of ASD risk gene prediction.

## Data Availability

Dataset and source code are available at https://github.com/Onebear-X/DeepASDPred is freely available.

## Abbreviations

ASD: Autism spectrum disorders
SVM: Support vector machine
LR: Logistic regression
RF: Random forest
CNN: Convolutional neural network
LSTM: Long short-term memory
ROC AUC: Area under the receiver operating characteristic curve
PR AUC: Area under the Precision-Recall curve
MCC: Mathews Correlation Coefficient
RPKM: Reads per kilobase of transcript per million mapped reads
PseAAC: Pseudo amino acid composition
PseKNC: Pseudo k-tuple nucleotide composition
ReLU: Rectifed linear unit
RNN: Recurrent neural network

## References

[1] Constantino JN, Zhang Y, Frazier T, Abbacchi AM, Law P (2010). Sibling recurrence and the genetic epidemiology of autism. Am J Psychiatry.

[2] Hallmayer J, Cleveland S, Torres A, Phillips J, Cohen B, Torigoe T, Miller J, Fedele A, Collins J, Smith K (2011). Genetic heritability and shared environmental factors among twin pairs with autism. Arch Gen Psychiatry.

[3] Ozonoff S, Young GS, Carter A, Messinger D, Yirmiya N, Zwaigenbaum L, Bryson S, Carver LJ, Constantino JN, Dobkins K (2011). Recurrence risk for autism spectrum disorders: a Baby Siblings Research Consortium study. Pediatrics.

[4] Sanders SJ, Murtha MT, Gupta AR, Murdoch JD, Raubeson MJ, Willsey AJ, Ercan-Sencicek AG, DiLullo NM, Parikshak NN, Stein JL (2012). De novo mutations revealed by whole-exome sequencing are strongly associated with autism. Nature.

[5] Iossifov I, O'Roak BJ, Sanders SJ, Ronemus M, Krumm N, Levy D, Stessman HA, Witherspoon KT, Vives L, Patterson KE (2014). The contribution of de novo coding mutations to autism spectrum disorder. Nature.

[6] Zhou J, Park CY, Theesfeld CL, Wong AK, Yuan Y, Scheckel C, Fak JJ, Funk J, Yao K, Tajima Y (2019). Whole-genome deep-learning analysis identifies contribution of noncoding mutations to autism risk. Nat Genet.

[7] Roundtree IA, Evans ME, Pan T, He C (2017). Dynamic RNA modifications in gene expression regulation. Cell.

[8] Jonkhout N, Tran J, Smith MA, Schonrock N, Mattick JS, Novoa EM (2017). The RNA modification landscape in human disease. RNA.

[9] Bruining H, Eijkemans MJ, Kas MJ, Curran SR, Vorstman JA, Bolton PF (2014). Behavioral signatures related to genetic disorders in autism. Mol Autism.

[10] Katuwal GJ, Cahill ND, Baum SA, Michael AM (2015). The predictive power of structural MRI in autism diagnosis. Annu Int Conf IEEE Eng Med Biol Soc.

[11] Xiao X, Fang H, Wu J, Xiao C, Xiao T, Qian L, Liang F, Xiao Z, Chu KK, Ke X (2017). Diagnostic model generated by MRI-derived brain features in toddlers with autism spectrum disorder. Autism Res.

[12] Ecker C, Bookheimer SY, Murphy DG (2015). Neuroimaging in autism spectrum disorder: brain structure and function across the lifespan. Lancet Neurol.

[13] Cogill S, Wang L (2016). Support vector machine model of developmental brain gene expression data for prioritization of autism risk gene candidates. Bioinformatics.

[14] Gok M (2019). A novel machine learning model to predict autism spectrum disorders risk gene. Neural Comput Appl.

[15] Wang J, Wang L (2020). Prediction and prioritization of autism-associated long non-coding RNAs using gene expression and sequence features. BMC Bioinform.

[16] Zhao Y, Zhao P, Liang H, Zhang X (2020). Identifying genes associated with autism spectrum disorders by random walk method with significance tests. IEEE Access.

[17] Hasan M, Ahamad MM, Aktar S, Moni MA: Early stage autism spectrum disorder detection of adults and toddlers using machine learning models. In*: 2021*. IEEE: 1–6.

[18] Lin Y, Yerukala Sathipati S, Ho SY (2021). Predicting the risk genes of autism spectrum disorders. Front Genet.

[19] Bradley AP (1997). The use of the area under the roc curve in the evaluation of machine learning algorithms. Pattern Recogn.

[20] Grau J, Grosse I, Keilwagen J (2015). PRROC: computing and visualizing precision-recall and receiver operating characteristic curves in R. Bioinformatics.

[21] Tang X, Zheng P, Li X, Wu H, Wei DQ, Liu Y, Huang G (2022). Deep6mAPred: A CNN and Bi-LSTM-based deep learning method for predicting DNA N6-methyladenosine sites across plant species. Methods.

[22] Chaste P, Leboyer M (2012). Autism risk factors: genes, environment, and gene-environment interactions. Dialogues Clin Neurosci.

[23] Abrahams BS, Arking DE, Campbell DB, Mefford HC, Morrow EM, Weiss LA, Menashe I, Wadkins T, Banerjee-Basu S, Packer A (2013). SFARI Gene 2.0: a community-driven knowledgebase for the autism spectrum disorders (ASDs). Mol Autism.

[24] Hawrylycz MJ, Lein ES, Guillozet-Bongaarts AL, Shen EH, Ng L, Miller JA, van de Lagemaat LN, Smith KA, Ebbert A, Riley ZL (2012). An anatomically comprehensive atlas of the adult human brain transcriptome. Nature.

[25] Harrow J, Frankish A, Gonzalez JM, Tapanari E, Diekhans M, Kokocinski F, Aken BL, Barrell D, Zadissa A, Searle S (2012). GENCODE: the reference human genome annotation for The ENCODE Project. Genome Res.

[26] Chou KC (2011). Some remarks on protein attribute prediction and pseudo amino acid composition. J Theor Biol.

[27] Chou KC (2005). Using amphiphilic pseudo amino acid composition to predict enzyme subfamily classes. Bioinformatics.

[28] Chen W, Lin H, Chou KC (2015). Pseudo nucleotide composition or PseKNC: an effective formulation for analyzing genomic sequences. Mol BioSyst.

[29] Liu B, Liu F, Wang X, Chen J, Fang L, Chou KC (2015). Pse-in-One: a web server for generating various modes of pseudo components of DNA, RNA, and protein sequences. Nucleic Acids Res.

[30] Mapleson D, Accinelli GG, Kettleborough G, Wright J, Clavijo BJ (2017). KAT: a K-mer analysis toolkit to quality control NGS datasets and genome assemblies. Bioinformatics.

[31] Matias Rodrigues JF, Schmidt TSB, Tackmann J, von Mering C (2017). MAPseq: highly efficient k-mer search with confidence estimates, for rRNA sequence analysis. Bioinformatics.

[32] Zhu-Hong Y, MengChu Z, Xin L, Shuai L (2017). Highly efficient framework for predicting interactions between proteins. IEEE Trans Cybern.

[33] Su ZD, Huang Y, Zhang ZY, Zhao YW, Wang D, Chen W, Chou KC, Lin H (2018). iLoc-lncRNA: predict the subcellular location of lncRNAs by incorporating octamer composition into general PseKNC. Bioinformatics.

[34] Wang JS, Zhang SL (2021). PA-PseU: An incremental passive-aggressive based method for identifying RNA pseudouridine sites via Chou's 5-steps rule. Chemometr Intell Lab.

[35] Yin. W, Kann. K, Yu. M, Schutze. H: Comparative study of CNN and RNN for natural language processing.

[36] Krizhevsky A, Sutskever I, Hinton GE (2017). ImageNet classification with deep convolutional neural networks. Commun ACM.

[37] Liu ZY, Xing JF, Chen W, Luan MW, Xie R, Huang J, Xie SQ, Xiao CL (2019). MDR: an integrative DNA N6-methyladenine and N4-methylcytosine modification database for Rosaceae. Horticult Res.

[38] Pearlmutter BA (1989). Learning state space trajectories in recurrent neural networks. Neural Comput.

[39] Vaswani A, Shazeer N, Parmar N, Uszkoreit J, Jones L, Gomez AN, Kaiser L, Polosukhin I: Attention Is All You Need. In: 31st Annual Conference on Neural Information Processing Systems (NIPS): Dec 04–09 2017; Long Beach, CA. 2017.

[40] Raffel C, Ellis DPWJA: Feed-forward networks with attention can solve some long-term memory problems. 2015, https://arxiv.org/abs/1512.08756.

[41] Lyall K, Croen L, Daniels J, Fallin MD, Ladd-Acosta C, Lee BK, Park BY, Snyder NW, Schendel D, Volk H (2017). The changing epidemiology of autism spectrum disorders. Annu Rev Public Health.

